# Indents

**Published:** 1908-12-15

**Authors:** 


### INDENTS.

☞ **Brief Articles of Technical or Scientific Value will receive notice and comment. Contributions invited.**

**Investment That Stands a High Heat.**

Pulverized Soapstone ........ 1 part.
Plumbago ........ 3 parts.
Asbestos, grade No. 3 ........ 5 parts.
Plaster of paris ........ 7 parts.

Mix thoroughly and sieve.—*Brief.*

**Perfect Refrigeration Without Pain.** To obtain perfect refrigeration without causing any discomfort to the patient during automization of ethyl chloride, surround the place of operation with tissue paper, leaving a suitable opening, and folding the paper in a fan shape. The flow of saliva will not inconvenience either patient or operator.—*American Journal.*

**Supporting Delicate Work While Polishing.** Fusible metal may be used to support delicate orthodontia and bridgework pieces during polishing. It is readily cast where needed, is strong and rigid and may be removed by boiling in water, and a final boil in nitric acid without risk to the work. Zinc phosphate cement is preferable in some cases, It is removed by boiling in nitric acid. —Trueman, in *Brief.*

**Stove Polish for Inlays.** The burning of fingers by the use of hydrofluoric acid makes me feel that this is an opportune time to remark that, instead of using an acid in cleaning the casts, I use carburet of iron, that is to say, the ordinary black stove polish; I take a small brush and apply the carburet of iron to my wax impression, and that gives me, on casting in a mold which has been allowed to cool, a cast cleaner than I can get with the subsequent use of any of the acids.—Dr. Nies, *Items of Interest.*

**To Prevent "Clacking."** To prevent the clacking of artificial dentures, Dr. Emile Limet recommends forming the upper back teeth of white vulcanite, molding it to represent as near as may be the porcelain teeth. This unpleasant noise a few patients wearing artificial dentures make during mastication has long been the *bete noire* of the prosthetic dentist. It was bitterly complained of when bone dentures were in use. No explanation of its cause has been generally accepted.—*Dental Cosmos*, October, 1908.

**Two Pins for Bicuspid Crowns.** I could never bring myself to understand the advisability of using a double pin in a bicuspid—that is to say, a pin with with both points of equal length. To insert that double pin in a bicuspid root it would be necessary to cut that bicuspid root in both directions to give ample space to put both ends in at the same time. That weakens the root; but if one pin is made considerably shorter than the other, you save a great deal of the tooth structure, and I think it is equally as strong.—Dr. Hamlet, *Items of Interest.*

**Finishing Gold Inlays.** I get the best result when I do the final grinding, and coarse disking, or stripping with the inlay well seated in the cavity before it is cemented. I grind, disc, or strip in a direction to draw the feather edge of gold toward the margins. If it is a proximo-occlusal inlay, after it is cut down to what it is desired, remove and polish the proximal surface, clean thoroughly and wash it with alcohol and dry. Roughen the cavity side of inlay by scratching with a sharp-pointed excavator to insure a better attachment of cement.—G. W. Dittmar, Chicago *Review.*

**Porcelain in Its Proper Place.** Porcelain has had its trial and has been condemned by many, and yet it has proved to be a godsend in proper hands and in the right place; therefore without doubt it must be placed on the list of standard filling materials. It has been run to earth by some, but it is a pleasure to announce that after careful investigation I find very few of the better class of practitioners unwilling to acknowledge that a sudden withdrawal of its use would be a step backward, and that its application, although it has been abused, has determined indisputable facts.—W. A. CAPON, in *Cosmos*.

**Malocclusion of Teeth.** As to the correction of malocclusion of the teeth, an appliance that is at the command of the dentist is often neglected. We have carbon paper or impression paper for determining whether or not the teeth occlude properly, and we neglect the use of that more than any one thing in our offices. All our fillings and crowns should be tested to see whether or not they are in proper occlusion. The occlusion may be bad on account of some change we have made, or on account of some change made by some of the teeth moving. Pyorrhea can be prevented, or the cure of it assisted, by the intelligent use of the carborundum wheel and sandpaper disk.—A. E. Royce, *Dental Surgeon*.

**Immediate Root Filling.** The rubber dam being applied, the contents of canal are removed mechanically, several applications of full strength sulphuric acid applied for two or three minutes, each being neutralized by saturated solution of bicarbonate of soda. Ten minutes of 5 percent. of formalin solution completes sterilizing, the canal being filled with some form of antiseptic material; in most cases the clinician uses a proprietary preparation, "Jodo-formagin," because of its power and convenience.

Results of ten years have been highly gratifying, not more than one in fifty being succeeded by soreness; and but two or three cases have ever been followed by return of septic conditions, so far as known.—F. W. Barbour, in *Dominion Journal.*

**Porcelain vs. Silicates.** I believe that many of us are using the silicate cement fillings, where we should really use the porcelain inlay. I have no doubt but that we are prone to so employ them because they are so much more convenient and rapid to insert in the cavities. As for myself, I am wedded to the porcelain inlay, and am putting in as many to-day as I have ever done. It is said that the porcelain inlays are going out. Not by any means. They are not going out. They will stand the stress of mastication in many cases where we do not know that the silicate cement will. I have had failures with the silicate cement fillings, although very few, indeed.—J. D. Patterson, *Western Dental Journal.*

**Use of an Assistant.** No operator should take time to pick up instruments, or to go to the laboratory for anything an assistant could do. The assistant should be well enough trained to know what was coming next. I do not expect to look for my forceps, or to look for a tool of any character when the operation comes; I expect my assistant will see that it is in my hand. I take the attitude that a man's time is worth whatever he thinks it is and he cannot afford to do what an assistant can do, who is paid from six to fifteen dollars a week. He cannot afford to do those things, as his time is worth too much. I keep two young ladies to do these things for me. I do not go to my laboratory; they take care of my vulcanizer when that is cared for, they do the polishing and do every essential that comes to hand, which their knowledge enables them to do. It is worth something to them to have the opportunity to do it, and it is worth more to me and to my patients that they are able to do it.—Dr. Noble, in *Western Journal.*

**A Pyorrhoea Treatment.** The following remedy is prepared for each case, and when ready for application is the consistency of a thick liquid. It is placed in the pockets by using small particles of absorbent cotton on a fine broach, afterwards covering the teeth and gums with a saturate alcoholic solution of the refined cubes of rosin used by violinists; this, evaporated by hot air, leaves a coating to keep moisture from the treated parts. The teeth and gums must be thoroughly dried with hot air before making the application. Formula—A minute particle of silver nitrate crystal, argyrol, carbolic acid, and a small portion of orthoform, well spatulated into a paste or thick liquid. This is a powerful antiseptic, and reduces the pain almost instantly.—Dr. Ella M. Hind, *The Dental Scrap Book*, October, 1908.

**Enlarging Pulp Canals.** The usual directions given for enlarging pulp canals by first using a small instrument, and then the next size larger and so on until the enlargement is satisfactory, is not mechanically the best way. From the beginning to the end of the operation the instruments used are always under strain. The inventor of the Morey pulp-canal drills suggested a better way. First use the largest that is intended to be used, and with it drill in as far as deemed prudent; then take the next size smaller, and so on until the operation is complete. By this latter plan the smaller and more delicate instruments have much less work to do, they are less liable to clog, they are free to follow slight deviations from a straight line, and the operator is better able to see what is going on. Although this idea was given to the profession more than a score of years ago, it is seldom quoted, and its mechanical advantages do not seem to have been appreciated. A trial of the two methods will demonstrate that the Morey plan favors smooth cutting, rapidity, freedom from accidental fracture of instruments, and a longer life to the cutting tool.—*Dental Brief.*

**Medical Graduate of 1908.** The medical schools of this country awarded this year 1,674 fewer diplomas than in 1907; and 2,602 less than in 1906, when the graduates numbered 25,204. This decrease is held to be a sign that practitioners are becoming more competent. A number of schools presumed to be of inferior standing were closed during the current year; whilst nearly all medical institutions of the first class are requiring their candidates to be college graduates. Dr. John Harper Long has declared that of the 160 medical schools in this country in 1907 fully one-half had "no moral right to exist"—which is putting things uncommonly strong; and that the majority of entering students are not properly equipped to understand what should be offered them in the newer pathology and etiology and in bacterial and physiological chemistry.—*Medical Times.*

**Hydrofluoric Acid; Be Careful When Using It.** I want to give you one little word of warning which I hope none of you will ever need. Dr. Taggart recommended to us hydrofluoric acid for cleaning the gold of cast inlays. I learned to-day that it is used for cleaning the castings of iron and steel. Workmen in foundries have learned to use it much diluted, and to protect their hands with rubber gloves. I have always known that it was a dangerous thing to handle, and I supposed that I was using it with the greatest caution, but found in a short time all the glassware in my laboratory was being ruined; glasses not nearer than four or five feet were clouded by the hydrofluoric acid fumes. Recently I injured myself with it and have been unable to do any work requiring the use of any force whatever since that time, and I dwell on this because all of the acid that reached my finger was by touching the cork; yet that tiny drop getting under the nail caused me intense pain all that night, and the wound required nine weeks to heal.—Dr. Ottolengui, *Items of Interest*, July, 1908, page 546.

**Disinfectant Mouth-Wash.** Dr. Wadsworth gives a formula of what he considers to be the best preparation:

| | |
|---|---|
| R—Sodium chlor. (c. p.) | ℥ss |
| Sodium bicarb. (c. p.), | gr. x. |
| Water (dist.), | ℥ij |
| Glycerin, | ℥j |
| Alcohol, | ℥v |
| Thymol, | ------ |
| Menthol, | aagr. j. |
| Oil of wintergreen, | gtt. iij |
| Oil of cinnamon, | gtt. ij |
| Oil of eucalyptus, | gtt. v. |
| Tr. cudbear, | ℥jss |
| Tr. rhatany, | ℥ss |
| Sig.—Dilute with water equal parts. | |

This formula may look formidable, but not one of its ingredients will detract from the good effect of the alcohol. —Dr. S. G. Perry, in August *Cosmos.*

**Rapid and Accurate Method of Making Matrices.** E. S. Gaylord showed a rapid and accurate method of obtaining matrices for inlays, substituting a medium weight China silk in lieu of gold-beaters' skin—as suggested by Dr. Emil Schreier, of Vienna—as a cradle support for gold or platinum foil. The silk having greater bulk, sustains the foil better than does the vellum. The procedure is as follows: The foil being cut sufficiently large to amply cover the prepared cavity, is placed between a fold of silk, then dipped in water, and with wet pellets of cotton, bibulous paper, or spunk, is quickly carried to the floor of the cavity and swaged to form without tear or puncture; then it is taken from the cavity, the silk is carefully removed, and the matrix returned for completion by again swaging with pellets of cotton, bibulous paper or spunk, which are this time used dry. The operation was completed by filling with wax or gum camphor for safe removal.—*Cosmos.*

**Zinc, Chlorid, Modified.** Zinc chloride coagulates albumin and in the process hydrochloric acid is liberated. For this reason the application of strong solutions is painful and should not be employed in deep cavities unless the irritating action of the agent is modified. This can be done to a marked degree by selecting alcohol and chloroform as the vehicle in which to make the solution. A useful formula is as follows:

| | |
|---|---|
| Zinci chloridi | gr.xx |
| Alcoholis | f.ʒiv. |
| Chloroform, q. s. ad | f℥i—M. |

Sig.—Apply to the cavity on a small pledget of cotton and gently evaporate to dryness.

NOTE.—If the zinc salt does not make a clear solution in the alcohol it indicates that some of the salt has been oxidized; the solution can be cleared by adding one drop of hydrochloric acid.—Dr. J. P. Buckley.

**Joining Electric Wires.** The following points must be remembered if it is desired to construct a joint that will hold, and not cause trouble through leakage, corrosion, or undue resistance to the current. Never cut the covering from a wire with the edge of the trimming knife toward the metal, as the slightest nick in the metal will cause the wire to break on bending. Use the knife in trimming the insulating material surrounding the wire as you would in sharpening a lead pencil, along the same plane as the wire. See that the material is clean in every respect before commencing to connect up and join. Never use an acid in soldering your joint, but use rosin or some suitable stick compound as sold at electrical stores. The following simple solution is excellent: Take wood alcohol and dissolve in it as much rosin as will make a varnish; apply this to the joint and then solder up. After soldering, draw over the joint the india rubber insulation, molding it closely to the wire, and finally well wrap with tape. It is very important that the joint be made water and moisture tight, otherwise changes may take place increasing the electrical resistance and fire risk.—H. Meredith Jones, *The Moving Picture World.*

**Tinfoil in Plate Work** The one thing above all others which I want to put stress upon is the application of tinfoil. By applying the tinfoil upon the model after it has been properly scraped, my claim is that the plate will fit very much better than if it were made on the plaster model, from the fact that it is made on a smooth, glassy surface, and consequently the suction is much stronger. Another claim which I make is that the plate, being made between tin dies, as it were, is very much more dense, consequently more healthful for the mouth, inasmuch as it will not absorb the secretions of the mouth to nearly so great an extent as when made on plaster. And because the plate has a glossy like surface next to the roof of the mouth, it will not irritate the mucous membrane and cause what is known as rubber sore mouth, as it will often do when made of plaster. In many, many cases of what has been known as rubber sore mouth, where the mouth has been almost like a piece of raw beefsteak, I have cured them with a plate made in this way.—Dr. S. C. G. Watkins, in *Items of Interest.*

**Sterilization of Dental Instruments.** In order to have dental instruments perfectly sterile, it is quite necessary to have them free from all traces of rust, particularly the kind of rust which makes little more than tarnish. The best way for keeping instruments bright and free from rust is to immerse them for some hours, or even for days, if one wishes, in a saturated solution of stannous chlorid. A basin of stannous chlorid crystals to which water is added from time to time can be kept in the office, and instruments can be laid in it from time to time. The stannous chlorid makes a soluble salt of the iron oxid, which causes the tarnish to vanish, and removes not only hiding-places, for minute bacteria, but also keeps the sharp edges of the instrument intact.

Boiling of instruments in a 1 percent. solution of sodium carbonate furnishes an excellent method of sterilization and one which practically fulfills all the indications, as it removes traces of organic matter at the same time that bacteria are destroyed. The addition of the sodium carbonate to the water is for the purpose of preventing oxidation which would tarnish the instruments and which would dull sharp edges. The boiling of instruments causes a good deal of special trouble and attention, and for that reason it is very often neglected.

Perhaps the most practical method for sterilization of instruments in the offices of very busy men is the carbolic acid and alcohol method. Instruments, even imperfectly cleansed, can be kept in a receptacle of carbolic acid, where they are practically sterilized and remain sterile. They may remain indefinitely in such a receptacle because the carbolic acid, a phenol, is closely related to the alcohols and does not attack metals. When instruments kept in carbolic acids are wanted for use they are picked out with a pair of forceps and put into another receptacle of alcohol. The alcohol instantly neutralizes the carbolic acid and removes it. If patients object to the odor of alcohol on instruments, the instruments can be dropped into another receptacle of sterilized water for a moment. For all of the smaller instruments it seems that this is the best practical method for sterilization in every-day office work.—R. T. Morris, *Dental Brief*.

**Amalgam Inlays.** Dr. H. W. C. Bodecker, of Berlin, Germany, advocates the use of amalgam inlays. The cavity for an amalgam inlay is prepared precisely as one would prepare a cavity for a gold inlay. Thin walls of enamel should be removed, the margins made smooth, and as far as possible, at right angles to the surface. He prepares cavities for taking the impression by the use of stones wherever they can be used. Two shapes only are needed, one slightly conical barrel shaped, of different diameter, and very small round stones, such as the gem cavity points. In most cases, he states, it is possible to shape proximo-occlusal cavities so that the inlay will be self-retentive, by running a fissure bur at an angle of thirty degrees across the buccal and lingual margins of the occlusal surface. For taking the impression he uses Detroit perfection impression material made into cones of various sizes. The point is softened and the cone pressed into the cavity. To confine the material when necessary, a thin copper band is placed around the tooth, or a thin piece of sheet steel which reaches well up to the gingival margins of the cavity is held against the adjacent tooth. The model is made of ordinary plaster to which a little salt or potassium nitrate has been added. When dry, this makes a model sufficiently hard to resist the pressure used in packing the amalgam. All excess plaster is removed from the model so that the margins of the cavity and the immediately adjacent surface of the tooth stand out clearly. He prefers a slow-setting amalgam for contour work, or when a number of models are to be filled at one time. The filling should be made with as much care as if the operation was performed in the mouth. The doctor contends that an amalgam inlay is denser and more uniform in density than an amalgam filling made in the mouth, because, as the cavity is more accessible on the model, greater and more equally distributed pressure can be brought to bear on the whole mass of amalgam, giving it greater hardness, and at the same time rendering it less liable to distortion. Furthermore, in the filling on the model, not only does crystallization take place, undisturbed, but all danger of having the material bitten out of shape is avoided. The final surfacing and polishing is far more easily and accurately done on the model than it can be in the mouth, especially is this true where an interproximal space is involved. The doctor notes that amalgam lacks the tenacity of gold, and therefore is liable to fracture when extended as a thin process along a slightly deepened fissure.—*Dental Review*, February 1908, page 91.

